# Lung adenocarcinoma in a patient with Lynch syndrome: a case report and literature review

**DOI:** 10.3389/fonc.2023.1193503

**Published:** 2023-10-13

**Authors:** Alan Hodges, Kai Sun, Tiffany G. Sheu, Eric H. Bernicker

**Affiliations:** ^1^ Texas A&M School of Medicine, Bryan, TX, United States; ^2^ Houston Methodist Research Institute, Center for Immunotherapy Research, Houston, TX, United States; ^3^ Houston Methodist Neal Cancer Center, Houston, TX, United States; ^4^ Department of Pathology and Genomic Medicine, Houston Methodist Hospital, Houston, TX, United States

**Keywords:** Lynch syndrome, NSCLC, MLH1, immune checkpoint therapy, ctDNA

## Abstract

This article presents a case of a 62-year-old Vietnamese woman with a history of Lynch syndrome (LS), who developed lung adenocarcinoma with *EGFR* L858R mutation. LS is an autosomal dominant cancer predisposition syndrome caused by a pathogenic germline variant in DNA mismatch repair genes, often leading to microsatellite instability. While LS is primarily associated with gastrointestinal, endometrial, ovarian, and urologic tract cancers, lung cancer accounts for less than 1% of LS-related cancers, with only six cases of LS-related lung cancer previously reported in the literature. The patient underwent multiple lines of treatment for her lung adenocarcinoma, including tyrosine kinase inhibitors, stereotactic body radiation therapy, pemetrexed and pembrolizumab, amivantamab, and fam-trastuzumab deruxtecan, but all resulted in only a partial response followed by a progressive disease. This case highlights the complex interplay of genetic cancer predisposition syndromes and the development of spontaneous driver mutations in the disease course and the subsequent management of tumors arising in these patients.

## 1 Introduction

Lynch syndrome (LS) is a genetically defined disease entity often associated with the clinical syndrome hereditary non-polyposis colon cancer, an autosomal dominant cancer predisposition syndrome. Patients with LS have an increased risk of a wide array of malignancies, most commonly gastrointestinal cancer, endometrial cancer, ovarian cancer, and urologic tract cancer (1). LS is caused by an autosomal dominant, pathogenic, germline variant in one of the DNA mismatch repair (MMR) genes (*MSH2*, *MLH1*, *MSH6*, or *PMS2)* or *EPCAM*, which leads to epigenetic silencing of *MSH2* (2). Pathogenic variants in the MMR genes are relatively frequent, with an estimated prevalence of 1 in 279 (3), and thus LS represents one of the most prevalent cancer predisposition syndromes (4).

The resulting deficiency in MMR due to LS gene variants results in the accumulation of errors throughout the genome, including in short, repeated, microsatellite regions, a phenomenon termed microsatellite instability high (MSI-H). MSI-H is a hallmark of tumors associated with LS (5), and LS contributes to a significant proportion of MSI-H tumors across tumor types (6). Notably, there is significant heterogeneity in MSI prevalence between tumor types in LS patients, with a high MSI prevalence observed in ureteral, colorectal, and ovarian tumors (100%, 98%, and 94%, respectively) and a low MSI prevalence in tumors such as renal and primary brain tumors (25% and 0%, respectively) (7, 8). This MSI prevalence heterogeneity may have important treatment implications for LS-related tumors, as MSI-H tumors are more likely to respond to immune checkpoint therapy (7, 9), whereas the role of IC therapies in microsatellite-stable disease in LS patients is less clear (10).

Lung cancer is the most frequently diagnosed cancer and leading cause of cancer death (11), with cigarette smoking contributing significantly to the prevalence of the disease. While driver mutations are identified in tumors of both smokers and non-smokers, driver mutations are widely prevalent in the disease of non-smokers, occurring in 70% and 95% in cohorts of NSCLC and lung adenocarcinoma, respectively (12, 13). Among driver mutations in NSCLC, epidermal growth factor receptor (*EGFR*) mutations are the most common (13). Targeting *EGFR* mutations with tyrosine kinase inhibitors (TKIs) has revolutionized the therapeutic landscape of metastatic NSCLC. However, many patients eventually progress despite the initial good response and will receive chemotherapy-based second-line treatment. Despite the immune checkpoint blockade showing promising results in the second-line treatment of metastatic NSCLC (14, 15), those with EGFR mutations are unlikely to respond to immune checkpoint blockade (16, 17).

Less than 1% of lung cancer is associated with LS, and screening for LS is not recommended. Only six cases of lung cancer arising in patients with LS have been reported. *EGFR* mutation in LS-related lung cancer is even a rarer reported event. Here we report a case of incidentally discovered lung adenocarcinoma developing in a patient with a previous diagnosis of LS. Additionally, we review the leterature on lung cancer related to LS and the subset of this population with *EGFR*-mutated tumors.

## 2 Case presentation

**Figure 1:**
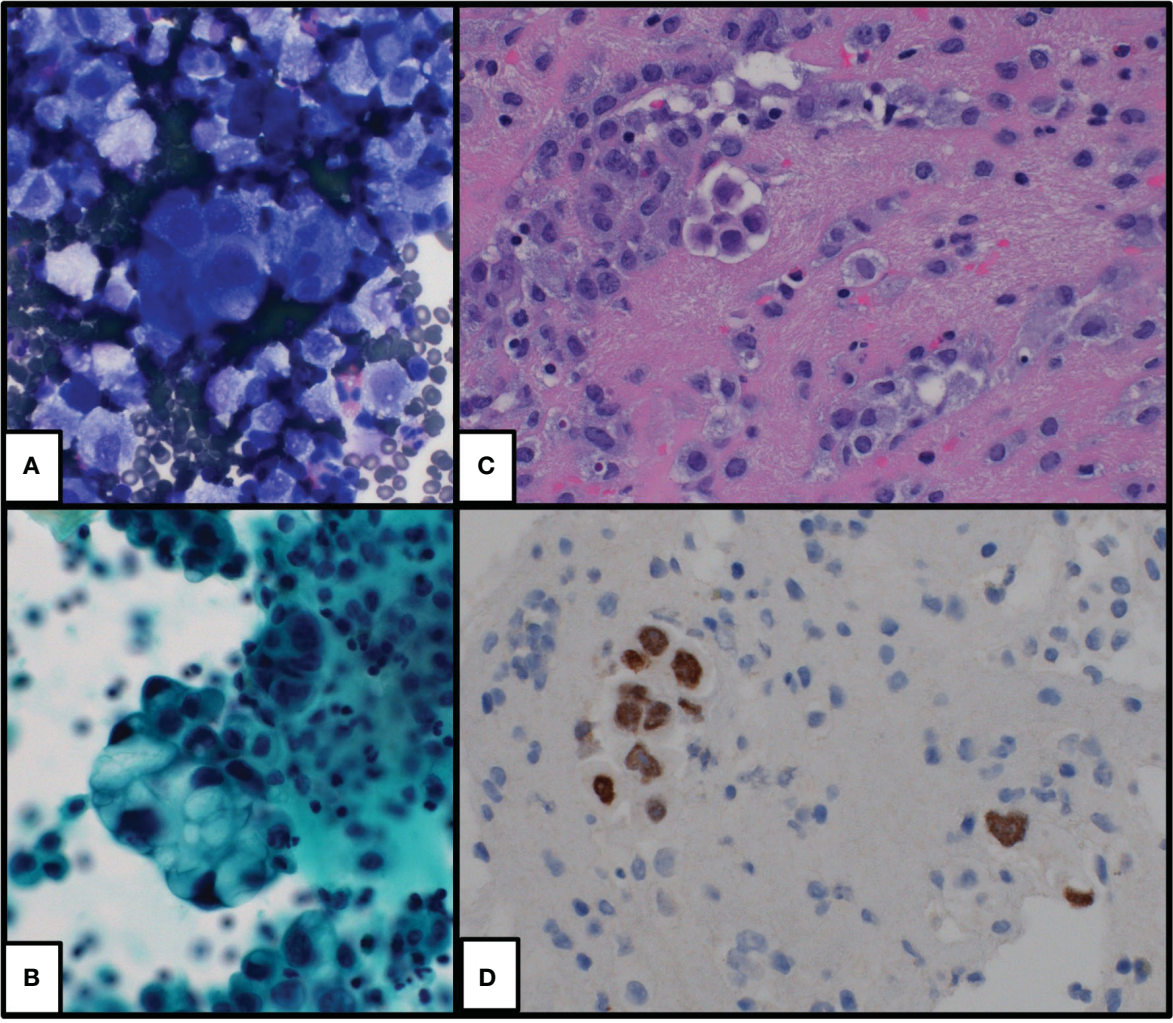
Cytologic preparations of initial pleural effusion which show groups and single malignant cells with morphologic and immunophenotypic findings supportive of lung adenocarcinoma. **(A)** DiffQuik-stained cytospin preparation at ×400, **(B)** Pap-stained cytospin preparation at ×400, **(C)** hematoxylin and eosin-stained cell block preparation at ×400, and **(D)** TTF1 immunohistochemical stain at ×400.

A 62-year-old Vietnamese woman, non-smoker, presented with dysphagia and odynophagia. She had a history of colon cancer that was treated with hemicolectomy and adjuvant chemotherapy at age 53 and stage I right upper lobe lung adenocarcinoma that was treated with lobectomy at age 57 in Vietnam. The family’s oncologic history was significant for colon cancer in her paternal grandmother and aunt. She was found to have left tongue squamous cell carcinoma by biopsy. During the staging of tongue squamous cell carcinoma, she was found to have one 1-cm right upper lobe nodule and one 1-cm left upper lobe nodule. The biopsy of the right upper lobe nodule revealed lung adenocarcinoma (**Figure 1**). Molecular testing showed *EGFR* L858R mutation—negative for *ALK* rearrangement, *BRAF*, and *MET* mutations. PD-L1 by 22C3 pharmDx was negative. A positron emission tomography/computed tomography scan showed moderate fluorodeoxyglucose uptake in both lesions, which are concerning for malignancy. Her family history was significant for colon cancer in her son at age 35 who was later found to carry the pathogenic germline *MLH1* variant. Due to the patient’s history of multiple malignancies and her family history, genetic testing was performed, which revealed a pathogenic germline *MLH1* variant, and the patient was subsequently diagnosed with LS.

**Figure 2:**
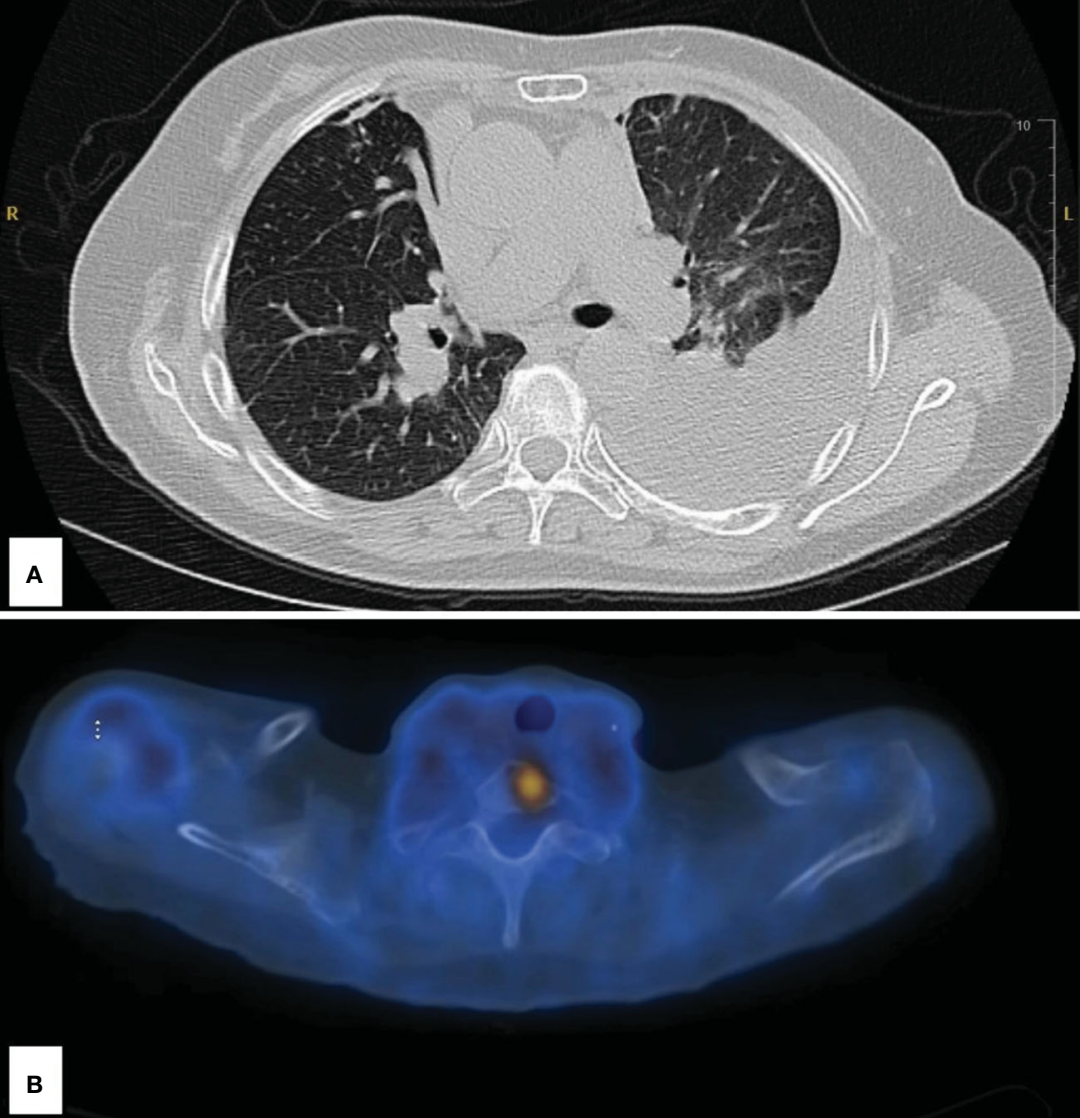
Imaging following disease progression at 2 years’ status post-stereotactic body radiation therapy. **(A)** Large left pleural effusion and left lower lobe atelectasis. **(B)** Abnormal fluorodeoxyglucose uptake in the upper spine; SUV of 5.5.

**Figure 3:**
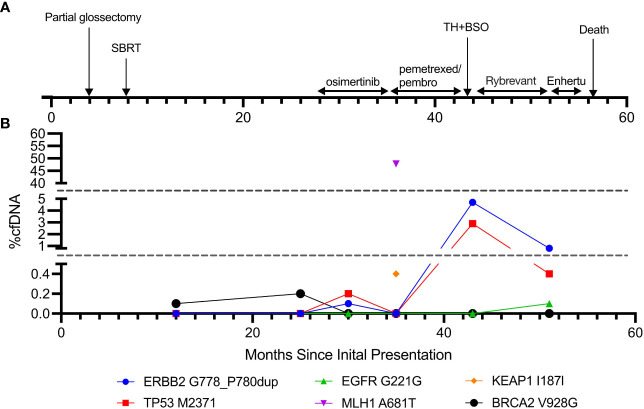
Timeline of the disease course and cfDNA trends. **(A)** Timeline of clinical course and treatment modalities. **(B)** cfDNA composition throughout the disease course.

She underwent left partial glossectomy and lymph node dissection for her stage II tongue squamous cell carcinoma. She then completed stereotactic body radiation therapy (SBRT, 50 Gy in five fractions) to both the left and right upper lobe lung lesions. However, she was found to have enlarged left lung nodules and new pleural effusion on surveillance scans 2 years after her SBRT treatment. The sampled pleural fluid contained cytologic features of adenocarcinoma. A ctDNA analysis by Guardant360 (18) was performed, but it only revealed BRCA2 variance of unknown significance. Given the *EGFR* mutation status on the initial tissue biopsy, she was started on osimertinib. She had stable disease for 6 months but then developed worsening pleural effusion and new bone lesions (**Figures 2A, B**). A repeat ctDNA was performed, and it showed *ERBB2* (G778_P780dup) and *TP53* (M237I) mutations at a very low frequency. Her treatment was switched to pemetrexed and pembrolizumab doublet. Platinum chemotherapy was omitted given her history of ischemic stroke and performance status. During the period following an initial partial response, she also received elective total hysterectomy and bilateral salpingo-oophorectomy for risk reduction. At 7 months after the initiation of the pemetrexed and pembrolizumab doublet, her disease progressed. The patient was then started on the EGFR/MET bispecific antibody amivantamab (Rybrevant), again showing partial response followed by a progressive disease after 7 months. Another ctDNA was conducted, and it revealed *ERBB2* and *TP53* mutations at low frequency. Notably, none of the ctDNA tests showed MSI-H disease but was significant for a *MLH1* A681T mutation at approximately 50% allele frequency (47.8%), which was likely from her LS-defining germline heterozygosity. The patient was subsequently started on fam-trastuzumab deruxtecan (Enhertu) for three cycles. At 1 month following the final cycle of fam-trastuzumab deruxtecan, the patient developed fatal acute respiratory failure secondary to pulmonary edema (**Figure 3**).

## 3 Discussion

We report the case of a patient with LS who was diagnosed with NSCLC with *EGFR* mutation and had a short response to EGFR-targeted tyrosine kinase inhibitor and subsequent immune checkpoint inhibitor. To our knowledge, this is the second reported case of NSCLC with *EGFR* mutation in patients with LS and the first reported case with a long-term clinical outcome.

LS is known as one of the most common forms of inherited cancer predisposition (3). Although LS is classically associated with increased risks of a wide array of malignancies (1), NSCLC is not traditionally believed to be one of them. Sun et al. (19) analyzed the germline mutational status of 1,179 paired samples of lung cancer tumor tissue and normal lung tissue, and only six of 1,179 (0.5%) patients were found to have germline MMR gene pathogenic variants. Takamochi et al. (20) analyzed the MSI status in 366 patient samples, and only one tumor sample was found to have MSI-H, and this patient had no background of LS. A larger study by Warth et al. (21) also confirmed low MSI-H frequency (0.8%) in patients with lung adenocarcinoma. These studies indicate that lung cancer with MMR germline pathogenic variants or MSI-H is a rare and sporadic event, and screening for LS in patients newly diagnosed with lung cancer will be low-yield and likely be cost-ineffective.

However, there have been several case reports of lung cancer that developed in patients with LS (**Table 1**). Including our case, all cases were diagnosed with lung adenocarcinoma with low or negative PD-L1 expression. MSH2 germline mutation was the most common mutation, followed by MLH1 mutation. In total, five of seven (71.4%) patients were found to have loss of MMR expression on tumor tissues; none of these five patients had *EGFR* mutation, and three out of these five patients had either remission or stable disease as the best response to second-line immune checkpoint inhibitors. Moreover, two of seven (28.6%) patients had tumors that harbored *EGFR* mutation, but neither tumor had loss of MMR expression. In the study by Warth et al. (21), two of four (50%) patients who had MSI-H tumors also harbored *EGFR* mutations, and in the study by Sun et al. (19), two of six (33.3%) patients who had germline MMR mutation harbored *EGFR* mutations. The prevalence of *EGFR* mutations in lung cancer patients with LS from the case reports and studies mentioned above is 35.3% (6/17), which is higher than that of 10%–20% observed in Europe and North America populations (28).

**Table 1:** Summary of patients with Lynch syndrome who developed lung cancer.

	Age/gender	Germline gene pathogenic variant	Other malignancies	Lung cancer histology	MMR (D/P)	MSI	PD-L1 level	*EGFR* mutation	Outcome
Canney et al. (22)	59/M	*MSH2*	Colon cancer	NSCLC	D^*^	Not reported	Not reported	Not reported	Not reported
Kawashima et al. (23)	68/M	*MSH2*	Colon, rectal, and prostate	NSCLC	D (loss of MSH2 and MSH6)	S	3%	No	Long-lasting response to nivolumab >22 months
Masuzawa et al. (24)	36/M	*MLH1*	None	NSCLC	D (loss of MLH1 and PMS2)	H	1%–24%	No	Long-lasting response to nivolumab >20 months
Nolan et al. (25)	64/M	*MSH2*	Colon and bladder	NSCLC	D (loss of MSH2)^†^	H	Not reported	No	Alive after surgery
Maccaroni et al. (26)	74/F	*MSH6*	Ovarian and rectal	NSCLC	D (loss of MSH6 on brain met)	H	Negative	No	4 months of SD on pembrolizumab, disease progression and death at 6 months
Hissong et al. (27)	66/F	*MSH2*	Endometrial	NSCLC	P	S	5%	*EGFR* L858A	Not reported
Our case	62/F	*MLH1*	Colon, tongue	NSCLC	Not reported	S	Negative	*EGFR* L858R	Deceased following failure of multiple lines of therapy

* Two lung lesions were identified: one had loss of MSH2 and MSH6 expression, and the other one was MMR proficient.

† Two lung lesions were identified: one had loss of MSH2 expression and was MSI-H, and the other one was MMR proficient.

Even though our patient harbored *EGFR* mutation, her response to osimertinib and immune checkpoint inhibitor with chemotherapy was short-lasting. Li et al. (29) reported the association between a stronger MLH1 expression and a higher *EGFR* mutation frequency. The authors predicted that the overexpression of MLH1 could be a potential marker for sensitivity to EGFR TKIs. If true, patients with LS with a loss of MLH1 expression would likely demonstrate a suboptimal response to EGFR TKIs. Moreover, although patients with LS are expected to have an increased response to immune checkpoint inhibitors, a decreased response to immune checkpoint inhibitors is observed in *EGFR*-mutated NSCLC at large (30) and likely contributed to the failure of immune checkpoint therapy in this patient. Furthermore, MMR deficiency in tumors arising in LS patients cannot be presumed, especially in non-typical LS tumor types, and therefore individualized testing of tumors may be warranted to guide the use of IC therapies in these patients.

This case also highlights the need to carefully weigh the decision to pursue risk reduction surgery, weighing the pathogenicity of different LS variants and the patient’s underlying comorbidities as recommended by the NCCN (31). Moreover, in patients with current and stable malignancies, the risks of exacerbating the disease through unrelated risk reduction surgeries must be considered (32, 33).

## 4 Conclusion

NSCLC is not among the malignancies that are commonly associated with LS. In patients with LS who developed NSCLC, *EGFR* mutation seems to be more prevalent and should be checked as in patients without LS. Despite the MSI-H status that is commonly seen in LS with an associated expected good response to immune checkpoint blockade, these patients with *EGFR* mutations and LS tend to have a poor response to immune checkpoint inhibitors.

## Data availability statement

The original contributions presented in the study are included in the article/supplementary material. Further inquiries can be directed to the corresponding author.

## Ethics statement

Written informed consent was obtained from the individual(s) for the publication of any potentially identifiable images or data included in this article.

## Author contributions

AH, KS, and EB each contributed to data gathering, analysis, and manuscript preparation. KS and EB contributed to the clinical care of the patient. TS provided the pathology analysis. All authors contributed to the article and approved the submitted version.
